# Exploratory Evaluation of Bezlotoxumab on Outcomes Associated With *Clostridioides difficile* Infection in MODIFY I/II Participants With Cancer

**DOI:** 10.1093/ofid/ofaa038

**Published:** 2020-01-31

**Authors:** Oliver A Cornely, Kathleen M Mullane, Thomas Birch, Sabine Hazan-Steinberg, Richard Nathan, Emilio Bouza, David P Calfee, Misoo Chung Ellison, Michael T Wong, Mary Beth Dorr

**Affiliations:** 1 Cologne Excellence Cluster on Cellular Stress Responses in Aging-Associated Diseases (CECAD), Department I of Internal Medicine, Clinical Trials Centre Cologne (ZKS Köln), University of Cologne, Cologne, Germany; 2 Section of Infectious Diseases, Department of Medicine, University of Chicago, Chicago, Illinois, USA; 3 Holy Name Medical Center, Teaneck, New Jersey, USA; 4 Ventura Clinical Trials, Ventura, California, USA; 5 Idaho Falls Infectious Diseases, Idaho Falls, Idaho, USA; 6 Department of Medicine, Universidad Complutense, Department of Microbiology and Infectious Diseases, Hospital Gregorio Maranon, CIBERES, Madrid, Spain; 7 Weill Cornell Medicine, New York, New York, USA; 8 Merck & Co., Inc., Kenilworth, New Jersey, USA

**Keywords:** cancer, *Clostridioides difficile*, CDI recurrence, hematologic malignancy, solid tumor

## Abstract

**Background:**

The incidence of *Clostridioides difficile* infection (CDI) is reportedly higher and the cure rate lower in individuals with cancer vs those without cancer. An exploratory post hoc analysis of the MODIFY I/II trials (NCT01241552/NCT01513239) investigated how bezlotoxumab affected the rate of CDI-related outcomes in participants with cancer.

**Methods:**

Participants received a single infusion of bezlotoxumab (10 mg/kg) or placebo during anti-CDI antibacterial treatment. A post hoc analysis of CDI-related outcomes was conducted in subgroups of MODIFY I/II participants with and without cancer.

**Results:**

Of 1554 participants in the modified intent-to-treat (mITT) population, 382 (24.6%) were diagnosed with cancer (bezlotoxumab 190, placebo 192). Of participants without cancer, 591 and 581 received bezlotoxumab and placebo, respectively. In the placebo group, initial clinical cure (ICC) was achieved by fewer cancer participants vs participants without cancer (71.9% vs 83.1%; absolute difference, –11.3%; 95% CI, –18.6% to –4.5%); however, CDI recurrence (rCDI) rates were similar in cancer (30.4%) and noncancer (34.0%) participants. In participants with cancer, bezlotoxumab treatment had no effect on ICC rate compared with placebo (76.8% vs 71.9%), but resulted in a statistically significant reduction in rCDI vs placebo (17.8% vs 30.4%; absolute difference, –12.6%; 95% CI, –22.5% to –2.7%).

**Conclusions:**

In this post hoc analysis of participants with cancer enrolled in MODIFY I/II, the rate of rCDI in bezlotoxumab-treated participants was lower than in placebo-treated participants. Additional studies are needed to confirm these results.

**Clinical Trial Registration:**

MODIFY I (NCT01241552), MODIFY II (NCT01513239).

The incidence and severity of *Clostridioides difficile* infection (CDI) have markedly increased during the last 2 decades [1–3]. Although in the majority of cases antibiotic treatment for primary CDI is successful, CDI recurrence (rCDI) occurs in ~25% of CDI cases, with a 38%–45% probability of subsequent recurrences in those who have a first recurrence [4–9].

The rate of hospital-onset CDI is twice as high in those with cancer compared with individuals hospitalized for other conditions [10, 11]. Furthermore, it has been reported that individuals with cancer have a lower cure rate and increased time to resolution of diarrhea (TTROD) following firstline anti-CDI antibiotics (vancomycin or fidaxomicin [12]) compared with those without cancer [13]. These associations are likely due to altered gut microbiota due to frequent exposure to broad-spectrum antibiotics, anticancer drugs, increased exposure to *C. difficile* during prolonged hospitalizations, or simply immunosuppression [11, 14–18]. Cancer has also been associated with an increased risk of in-hospital mortality in individuals with CDI and is an independent risk factor for rCDI [19, 20]. There is a need for novel therapies to prevent recurrence in this vulnerable population.

Bezlotoxumab (MK-6072) is a fully human monoclonal antibody against *C. difficile* toxin B that has been approved by the US Food and Drug Administration and the European Medicines Agency to prevent rCDI in adults receiving anti-CDI antibiotics and are at high risk of rCDI [21, 22]. In the MODIFY I and II Phase 3 trials, a single 10-mg/kg infusion of bezlotoxumab, alone or in combination with actoxumab, reduced rCDI over 12 weeks compared with placebo (absolute reduction, 10%; relative reduction, 38%) [23]. Additional analyses showed that participants with ≥1 risk factor for rCDI (age ≥65 years; history of CDI in previous 6 months; compromised immunity; severe CDI [Zar score ≥2]; ribotype 027, 078, or 244) had more substantial reductions in rCDI (absolute reduction, 16%; relative reduction, 43%) and participants with ≥3 risk factors for rCDI had the greatest extent of reduction (absolute reduction, 24.8%; relative reduction, 54%) [24].

Using pooled data from MODIFY I/II, this post hoc exploratory analysis investigated how treatment with bezlotoxumab affected the rate of rCDI and CDI-related outcomes in participants with cancer. To confirm the observations of a previous analysis that reported differences in CDI-related outcomes in patients with cancer vs with those without cancer [13], data from the MODIFY I/II placebo group were compared.

## METHODS

### Study Design

MODIFY I (NCT01241552) and MODIFY II (NCT01513239) were randomized, double-blind, placebo-controlled, multicenter, phase 3 trials that were conducted from November 1, 2011, to May 22, 2015, across 322 sites in 30 countries [23]. The trial participants were adults with primary or recurrent CDI who were receiving anti-CDI antibiotics (metronidazole, vancomycin, or fidaxomicin, chosen by the treating physician) with a planned 10–14-day course [23]. CDI was defined as diarrhea (≥3 unformed bowel movement [types 5–7 on the Bristol Stool Scale] [25] in 24 hours) associated with a positive local laboratory stool test result for toxigenic *C. difficile*. Eligible participants were randomly allocated in a 1:1:1:1 ratio to receive a single 60-minute infusion (received on study day 1) of bezlotoxumab alone (10 mg/kg), actoxumab and bezlotoxumab (10 mg/kg each), actoxumab alone (10 mg/kg, MODIFY I only), or placebo (0.9% saline) [23] while they continued to receive anti-CDI antibiotics. Participants recorded their unformed bowel movements until 80–90 days postinfusion and were instructed to collect a stool sample and return to the clinic if they experienced a return of diarrhea. Participants with active cancer and who were randomized to receive bezlotoxumab alone or placebo were included in the “cancer” subgroup of this post hoc analysis. Participants with active cancer were identified based on a review of conditions reported on the medical history case report form for malignant solid tumors and hematologic malignancies that were identified by the investigator as ongoing at the time of randomization.

MODIFY I and II were conducted in accordance with Good Clinical Practice guidelines and the provisions of the Declaration of Helsinki. The protocols and amendments were approved by the institutional review board or independent ethics committee at each study site. Written informed consent was provided by all participants before the trial began.

### End Points and Analysis Populations

Initial clinical cure (ICC) was estimated in the modified intent-to-treat (mITT) population and was defined as no diarrhea on the 2 consecutive days after completion of anti-CDI antibiotics administered for ≤16 calendar days. The initial clinical response was imputed as clinical failure if antibiotics were administered for >16 days. The mITT population included all randomized participants in the overall population of the MODIFY I/II trials who received study infusion (bezlotoxumab or placebo), had a positive stool test at baseline for toxigenic *C. difficile*, and were receiving anti-CDI antibiotics at the time of or within 1 day after randomization. In participants who achieved ICC (clinical cure population), rCDI was assessed and defined as a new episode of diarrhea (≥3 unformed bowel movements within 24 hours), associated with a positive local or central laboratory stool test for toxigenic *C. difficile* within 12 weeks after infusion of study medication [23]. Sustained clinical cure (SCC) was defined as ICC of the baseline episode of *C. difficile* infection and no recurrent infection through 12 weeks. TTROD was defined as the time in days until the end of diarrhea during the baseline CDI episode (ie, time to the first of 2 days with ≤2 loose stools in 24 hours). Rehospitalization within 30 days was assessed in participants who were inpatients at the time of randomization, were subsequently discharged, and then were rehospitalized within 30 days of discharge. Rehospitalizations were characterized as CDI-associated if due to rCDI or if rCDI occurred during rehospitalization. Adverse events (AEs), all-cause mortality at 12 weeks, and time to death were assessed in the all patients as treated (APaT) population, which included all randomized participants who received an infusion of study medication.

### Statistical Analysis

The baseline demographics and clinical characteristics of participants included in the mITT population were summarized descriptively unless otherwise specified for each treatment group by cancer and no-cancer subgroups. The rates of ICC, rCDI, and SCC and rate differences between the bezlotoxumab and placebo treatment groups and their 95% confidence intervals (CIs), by cancer and no-cancer subgroups, were calculated based on Miettinen and Nurminen’s method [26]. The nonparametric Kaplan-Meier method was used to estimate the time to rCDI and TTROD and time to death for each of the treatment groups by cancer and no-cancer subgroups.

Other outcomes and AEs of interest during the 12-week follow-up period were summarized descriptively using frequencies and percentages for each treatment group by cancer- and no-cancer subgroups.

## RESULTS

### Baseline Demographics and Clinical Characteristics

The integrated mITT population from MODIFY I/II consisted of 1554 participants, of whom 781 (50.3%) received bezlotoxumab and 773 (49.7%) received placebo. In total, 382 (24.6%) participants had cancer, including 107 (6.9%) with hematologic malignancy and 290 (18.7%) with solid tumors. Within the cancer subgroup, 190 (49.7%) and 192 (50.3%) participants were treated with bezlotoxumab and placebo, respectively (Table 1; Supplementary Figure 1). Of the 1172 participants with no cancer, 591 (50.4%) and 581 (49.6%) were randomized to receive bezlotoxumab and placebo, respectively. The proportions of participants with a solid tumor vs a hematologic malignancy were similar between the bezlotoxumab (solid tumor, 75.3%; hematological malignancy, 27.9%) and placebo (solid tumor, 76.6%; hematological malignancy, 28.1%) groups (Table 1).

**Table 1.** Demographics and Clinical Characteristics (mITT Population)

	Bezlotoxumab		Placebo	
	Cancer (n = 190), No. (%)	No Cancer (n = 591), No. (%)	Cancer (n = 192), No. (%)	No Cancer (n = 581), No. (%)
Clinical characteristics				
Inpatient at randomization	149 (78.4)	381 (64.5)	144 (75.0)	376 (64.7)
Female	98 (51.6)	344 (58.2)	99 (51.6)	350 (60.2)
Mean age (SD), y	65.0 (16.0)	60.8 (18.5)	67.0 (12.9)	62.4 (18.6)
Age ≥65 y	110 (57.9)	280 (47.4)	114 (59.4)	291 (50.1)
Primary CDI	126 (66.3)	394 (66.7)	124 (64.6)	374 (64.4)
≥1 CDI episodes in past 6 mo	51 (26.8)	165 (27.9)	48 (25.0)	171 (29.4)
1 previous CDI episode ever	34 (17.9)	116 (19.6)	26 (13.5)	106 (18.2)
≥2 previous CDI episodes ever	23 (12.6)	77 (13.1)	32 (17.6)	94 (16.4)
Severe CDI (Zar score ≥2)^a,b^	37 (19.5)	85 (14.4)	44 (22.9)	81 (13.9)
Immunocompromised^c^	78 (41.1)	100 (16.9)	68 (35.4)	85 (14.6)
Chemotherapy within 30 d before study randomization	54 (28.4)	39 (6.6)	39 (20.3)	28 (4.8)
Chemotherapy during 12-wk follow-up	59 (31.1)	46 (7.8)	51 (26.6)	34 (5.9)
Antibiotic during CDI treatment^d^	91 (47.9)	155 (26.2)	81 (42.2)	195 (33.6)
Antibiotic after CDI treatment^d^	86 (45.3)	160 (27.1)	69 (35.9)	155 (26.7)
≥1 predefined risk factors^e^	170 (89.5)	422 (71.4)	157 (81.8)	426 (73.3) 205 (35.3)
≥2 predefined risk factors^e^	93 (48.9)	216 (36.5)	104 (54.2)	
Charlson Comorbidity Index ≥3	133 (70.0)	186 (31.5)	137 (71.4)	166 (28.6)
Albumin <2.5 g/dL	31 (16.3)	70 (11.8)	37 (19.3)	66 (11.4)
Anti-CDI antibiotic				
Metronidazole	97 (51.1)	282 (47.7)	85 (44.3)	289 (49.7)
Vancomycin	85 (44.7)	287 (48.6)	98 (51.0)	275 (47.3)
Fidaxomicin	8 (4.2)	22 (3.7)	9 (4.7)	17 (2.9)
PCR ribotype^f^				
No. of participants with a positive stool	125	365	127	359
027, 078, or 244 strain	33 (26.4)	69 (18.9)	40 (31.5)	75 (20.9)
027 strain	29 (23.2)	60 (16.4)	35 (27.6)	65 (18.1)
Cancer type				
Hematologic malignancy	53 (27.9)	N/A	54 (28.1)	N/A
Received a stem cell transplant	8 (15.1)	N/A	10 (18.5)	N/A
Solid tumor	143 (75.3)	N/A	147 (76.6)	N/A

Abbreviations: CDI, *Clostridioides difficile* infection; mITT, modified intent-to-treat; N/A, not applicable; PCR, polymerase chain reaction; WBC, white blood cell.

^a^Prespecified risk factor.

^b^Based on the following: (1) age >60 years (1 point); (2) body temperature >38.3°C (>100°F; 1 point); (3) albumin level <2.5 g/dL (1 point); (4) peripheral WBC count >15 000 cells/mm^3^ within 48 hours (1 point); (5) endoscopic evidence of pseudomembranous colitis (2 points); and (6) treatment in an intensive care unit (2 points).

^c^Defined on the basis of a participant’s medical history or use of immunosuppressive therapy.

^d^Systemically bioavailable antibiotics not used to treat CDI.

^e^Predefined risk factors for recurrence of CDI: CDI history in the past 6 months, severe CDI at baseline (Zar score ≥2), age ≥65 years, CDI due to hypervirulent strain (ribotypes 027, 078, or 244), and/or immunocompromised.

^f^Denominator is participants in the mITT population with a positive culture.

Participants in the cancer subgroup were more likely to have ≥1 predefined risk factor for CDI compared with the no-cancer subgroup (bezlotoxumab, 89.5% vs 71.4%, respectively; placebo, 81.8% vs 73.3%), including a higher proportion of elderly participants (≥65 years: bezlotoxumab, 57.9% vs 47.4%, respectively; placebo, 59.4% vs 50.1%), a higher proportion with severe CDI (bezlotoxumab, 19.5% vs 14.4%, respectively; placebo, 22.9% vs 13.9%), and a higher proportion of CDI due to a hypervirulent strain (bezlotoxumab, 26.4% vs 18.9%, respectively; placebo, 31.5% vs 20.9%) (Table 1).

In both treatment groups, participants with cancer were more likely to discontinue from the study before the end of the 12-week follow-up period compared with participants without cancer (bezlotoxumab group, 18.9% vs 12.7%; placebo group, 23.4% vs 13.9%, respectively). The most frequent reason for premature study discontinuation was death (Supplementary Figure 1).

### Assessment of Potential Impact of Cancer as a Comorbidity on CDI-Related Outcomes

**Figure 1.**
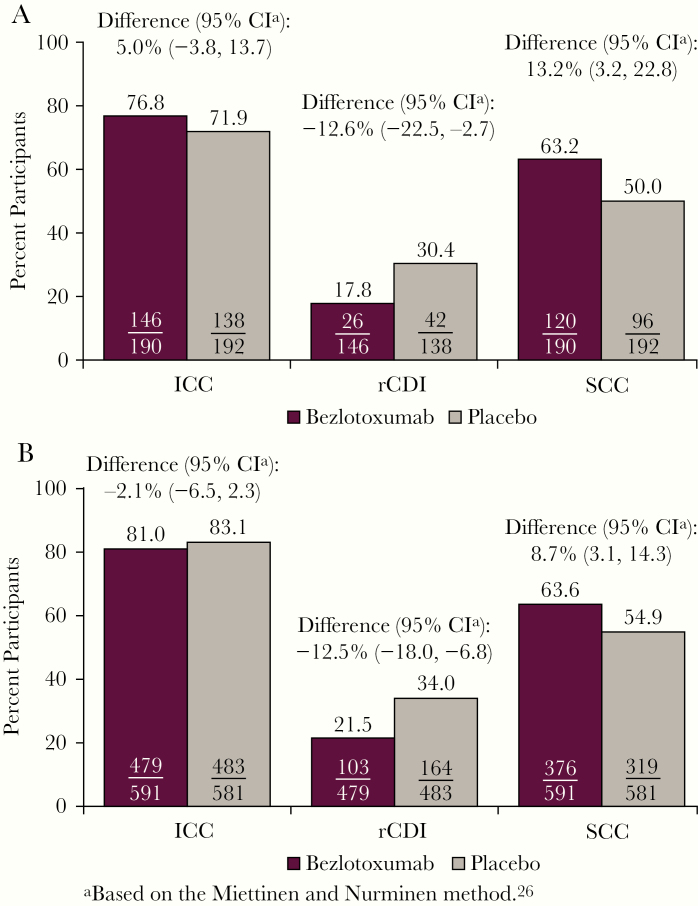
Proportion of participants with ICC, rCDI, and SCC stratified by treatment group in participants (A) with a cancer diagnosis and (B) without a cancer diagnosis. Abbreviations: CDI, *Clostridioides difficile* infection; CI, confidence interval; ICC, initial clinical cure; rCDI, recurrent *Clostridioides difficile* infection; SCC, sustained clinical cure.

In the placebo group, the ICC rate was lower in participants with cancer vs those without cancer (71.9% vs 83.1%) (Figure 1), and a statistical difference was observed (absolute difference, –11.3%; 95% CI, –18.6% to –4.5%). The TTROD was longer for the cancer subgroup compared with the no-cancer subgroup, but this trend was not significant (Supplementary Figure 3). More than 80% of participants had resolved their diarrhea within 7 days of study infusion.

There was no difference in the rate of rCDI between placebo participants with cancer (30.4%) vs those without cancer (34.0%) (Figure 1). Moreover, there was no difference in the SCC rate (cancer, 50.0%; no cancer, 54.9%). However, among participants receiving placebo who experienced a recurrence during the 12-week follow-up period, a higher percentage of patients with cancer (19.0%) had a severe episode compared with patients without cancer (7.3%) (Supplementary Table 2).

While there was no difference in the 30-day CDI-associated hospital readmission rate in placebo participants with cancer (11.1%) compared with those without cancer (11.2%) (Figure 3), the overall rate of serious AEs (SAEs) during the 12-week follow-up period was higher in participants with cancer compared with participants without cancer (45.6% vs 28.4%) (Table 2). Similarly, the proportion of placebo participants with cancer who died within 90 days of study enrollment was significantly higher compared with those without cancer (cancer: Kaplan-Meier [KM] estimate, 15.5%; 95% CI, 10.2% to 30.8%; no cancer: KM estimate, 5.5%; 95% CI, 3.6% to 7.4%) (Supplementary Figure 4).

**Table 2.** Adverse Event Summary (APaT Population)

	Bezlotoxumab		Placebo	
	Cancer, No. (%)	No Cancer, No. (%)	Cancer, No. (%)	No Cancer, No. (%)
Participants in population	191	595	193	588
During the 24 h after infusion				
Infusion-specific adverse reaction^a^	7 (3.7)	24 (4.0)	8 (4.1)	16 (2.7)
Discontinued infusion due to adverse reaction	0 (0.0)	1 (0.2)	0 (0.0)	0 (0.0)
During the 4 wk after infusion				
With ≥1 AEs	128 (67.0)	357 (60.0)	131 (67.9)	347 (59.0)
With drug-related AEs^b^	19 (9.9)	40 (6.7)	10 (5.2)	36 (6.1)
With serious AEs	55 (28.8)	101 (17.0)	58 (30.1)	109 (18.5)
With serious drug-related AEs	2 (1.0)	1 (0.2)	0 (0.0)	0 (0.0)
Who died^c^	9 (4.7)	18 (3.0)	13 (6.7)	14 (2.4)
Most common AE^d^				
Abdominal pain	12 (6.3)	22 (3.7)	11 (5.7)	23 (3.9)
Diarrhea	12 (6.3)	35 (5.9)	10 (5.2)	35 (6.0)
Nausea	12 (6.3)	40 (6.7)	13 (6.7)	26 (4.4)
Vomiting	11 (5.8)	20 (3.4)	5 (2.6)	16 (2.7)
Pyrexia	12 (6.3)	24 (4.0)	6 (3.1)	21 (3.6)
Urinary tract infection	12 (6.3)	20 (3.4)	10 (5.2)	25 (4.3)
Headache	7 (3.7)	28 (4.7)	6 (3.1)	18 (3.1)
During the 12 wk after infection				
Serious AE	80 (41.9)	151 (25.4)	88 (45.6)	167 (28.4)
With sepsis^e^	16 (8.4)	18 (3.0)	14 (7.3)	31 (5.3)
Who died^e^	20 (10.5)	34 (5.7)	28 (14.5)	31 (5.3)

Abbreviations: AE, adverse event; APaT, all patients as treated.

^a^AEs reported on the day of or day after infusion that were assessed by the investigator to be related to the study infusion. The investigator was unaware of the study group assignments.

^b^Assessed by the investigator to be related to the drug.

^c^Mortality was estimated at 30-day follow-up.

^d^Incidence ≥4% in either treatment group reported during the first 4 weeks after infusion.

^e^Sepsis and mortality were estimated at 90-day follow-up.

### Assessment of Potential Bezlotoxumab Impact on CDI-Related Outcomes in Participants With Cancer

**Figure 2.**
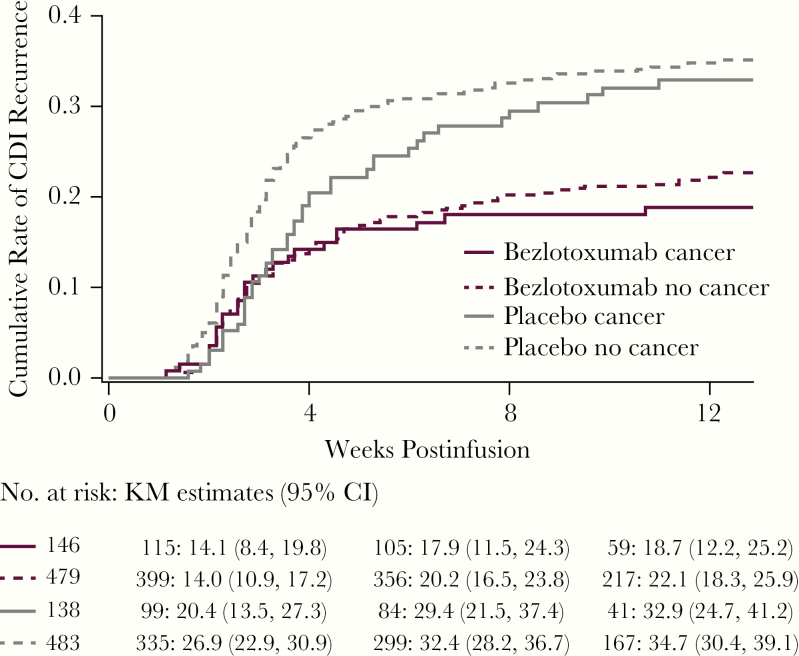
Time to rCDI stratified using the log-rank test by treatment group in participants with and without a cancer diagnosis (mITT population). The start date of rCDI was defined as the first day of a new episode of diarrhea. Time to event was right-censored at the date of the last stool record for any participants who were lost to follow-up before recurrence of CDI. Participants who completed the 12-week follow-up period without any reported episode of rCDI were censored at the date of the last completed stool record. For any participants who failed to achieve ICC for the baseline *C. difficile* episode, time to event was right-censored at the date of infusion of study medication. Abbreviations: CDI, *Clostridioides difficile* infection; CI, confidence interval; ICC, initial clinical cure; KM, Kaplan-Meier; mITT, modified intent-to-treat; rCDI, recurrent *Clostridioides difficile* infection.

The ICC rate in bezlotoxumab-treated cancer participants did not differ from that of placebo-treated cancer participants (76.8% vs 71.9%, respectively; absolute difference, 5.0%; 95% CI, –3.8% to 13.7%) (Figure 1). In contrast, a lower percentage of cancer participants treated with bezlotoxumab vs placebo experienced rCDI within 12 weeks of infusion of study medication (17.8% vs 30.4%, respectively; absolute difference, –12.6%; 95% CI, –22.5% to –2.7%; number needed to treat = 8). The cumulative rate of rCDI over the 12-week follow-up period, estimated via the nonparametric Kaplan-Meier method, was lower in bezlotoxumab-treated participants vs placebo; however, this difference was not statistically significant (bezlotoxumab: KM estimate, 18.7%; 95% CI, 12.2% to 25.2%; placebo: KM estimate, 32.9%; 95% CI, 24.7% to 41.2%) (Figure 2).

When stratified by solid tumor and hematologic malignancy, a smaller percentage of participants who received bezlotoxumab: 20.4%, placebo: 29.5%, absolute difference: –9.2%, 95% CI, –20.7% to 2.5%; hematologic malignancy: bezloloxumab: 14.0%, placebo: 33.3%, absolute difference: –19.4%; 95% CI, –38.1% to –0.7%) (Supplementary Figure 2A and B). In the Kaplan-Meier analyses, the cumulative rate of rCDI in bezlotoxumab participants with hematologic malignancy or solid tumor was lower vs placebo-treated groups (hematologic malignancy: bezlotoxumab 14.6%, placebo 35.1%; solid tumor: bezlotoxumab 21.4%, placebo 32.3%); however, the difference was not statistically significant in either group.

Participants receiving bezlotoxumab had higher rates of SCC compared with placebo (63.2% vs 50.0%, respectively; absolute difference, 13.2%; 95% CI, 3.2% to 22.8%) (Figure 1). Of the participants with cancer who experienced rCDI during the study, fewer bezlotoxumab-treated participants had a recurrent episode that was classified as severe (Zar score ≥2) compared with those who received placebo (3.8% vs 19.0%, respectively) (Supplementary Table 2). A higher proportion of bezlotoxumab-treated participants resolved the rCDI episode in ≤2 days (bezlotoxumab, 57.7%; placebo, 42.9%). Additionally, fewer bezlotoxumab-treated participants received a new course of antibiotic treatment (vancomycin, metronidazole, or fidaxomicin) for the rCDI episode (bezlotoxumab, 42.3%; placebo, 71.4%).

**Figure 3.**
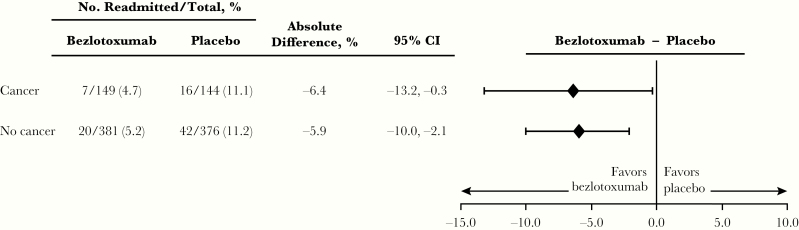
Proportion of participants with CDI-associated readmissions within 30 days of hospital discharge (among those hospitalized at the time of randomization). Abbreviations: CDI, *Clostridioides difficile* infection; CI, confidence interval.

The rate of CDI-associated 30-day rehospitalizations was lower in participants receiving bezlotoxumab compared with placebo (4.7% vs 11.1%; absolute difference, –6.4%; 95% CI, –13.2% to –0.3%) (Figure 3). Similar results were also observed in the solid tumor and hematologic malignancy subgroups (5.5% vs 10.2% and 4.3% vs 11.6%, respectively) (Supplementary Table 3).

The adverse event profile in participants with cancer was broadly similar in the bezlotoxumab and placebo groups (Table 2). However, the 90-day cumulative mortality rate was lower in those treated with bezlotoxumab compared with placebo, although this difference did not reach statistical significance (bezlotoxumab: KM estimate, 10.6%; 95% CI, 6.1% to 15.1%; placebo: KM estimate: 15.5%; 95% CI, 10.2% to 20.8%, respectively) (Supplementary Figure 4). Mortality rates by day 90 after bezlotoxumab treatment compared with placebo in the solid tumor and hematologic malignancy subgroups were 10.5% vs 15.6% and 9.3% vs 14.5%, respectively (Supplementary Table 3).

## Discussion

This post hoc analysis, which used pooled data from the MODIFY I/II trials, evaluated how treatment with bezlotoxumab affected the rate of rCDI and CDI-related outcomes in participants with and without cancer as a comorbid condition. A comparison between participants with cancer and those without cancer was also undertaken using data from the placebo group to confirm previous findings that showed that some CDI-related outcomes are worse in patients with cancer [13].

In the placebo-treated group of MODIFY I/II, significantly fewer participants in the cancer group achieved ICC compared with participants without cancer (71.9% vs 83.1%, respectively), consistent with a previous report [13]. Moreover, the ICC rate observed in placebo-treated MODIFY I/II participants with cancer was comparable with that reported in participants with cancer in the vancomycin arm of 2 phase 3 randomized trials of fidaxomicin vs vancomycin for *C. difficile*–associated diarrhea (71.9% and 74.0%, respectively), and, as in this analysis, these rates were lower than in the noncancer subgroup (83.1% and 88.7%, respectively) [13]. Similarly, the TTROD tended to be longer in placebo-treated participants with cancer compared with those without cancer; however, the difference was not as pronounced as previously reported. This is likely because approximately half of participants in the MODIFY trials had resolved their diarrhea by the day of study infusion in both subgroups due to the anti-CDI antibiotic that had been given on average for 3 days before infusion. The rates of rCDI and SCC in the placebo group were similar in both the cancer and no-cancer subgroups.

In participants with cancer, bezlotoxumab treatment had no effect on the ICC rate compared with placebo; however, as the mode of action of bezlotoxumab consists of binding to and neutralizing *C. difficile* toxin B, resulting in the prevention of rCDI, a significant effect on the ICC rate was not expected. The rate of rCDI in participants receiving bezlotoxumab was lower compared with placebo participants. The time to rCDI over the 12-week follow-up period was also lower in the bezlotoxumab treatment group compared with placebo; however, the difference was not statistically significant, which is likely due to the relatively small sample size. Compared with placebo, and consistent with the observed lower rates of rCDI, bezlotoxumab-treated participants with cancer experienced higher rates of SCC and lower rates of CDI-associated 30-day rehospitalization. Interestingly, among participants with cancer who experienced rCDI during the 12-week follow-up period, fewer episodes were severe (Zar score ≥2), a larger proportion were of short duration (≤2 days), and fewer were treated with a new course of antibiotics in the bezlotoxumab group compared with placebo. Additional subgroup analyses revealed a nonsignificant trend for a lower cumulative rate of recurrent CDI in participants with hematologic malignancy or solid tumor.

As expected, the 12-week all-cause mortality rate was higher in participants with cancer compared with participants without cancer in both treatment groups. There was a nonsignificant trend for a lower cumulative mortality rate at 90 days in bezlotoxumab-treated participants with cancer (10.5%) compared with placebo (14.5%).

In this post hoc analysis of the MODIFY trials, rCDI was experienced by 17.8% of bezlotoxumab-treated participants and 30.4% of placebo-treated participants with cancer as a comorbid condition. These cancer patients were more likely to have at least 1 risk factor for rCDI compared with those without cancer. These findings are consistent with previous research of individuals with cancer and CDI [20, 27] and support the need for effective treatments to prevent rCDI in these populations. The underlying malignancy, impaired immune response, and exposure to chemotherapy experienced by individuals with cancer may contribute to the incidence of CDI and subsequent recurrences [28]. Given the immunocompromised status of many individuals with cancer, treatment frequently includes concomitant systemic antimicrobial therapy, which further increases their risk for CDI and rCDI [29].

As this study was not specifically designed and statistically powered to evaluate the effect of bezlotoxumab in participants with cancer, there are several limitations that should be considered when interpreting results from this subgroup analysis. The analyses were retrospective and included a relatively small population of participants with cancer. The cancer subgroup was imbalanced compared with the noncancer subgroup with regards to participant numbers, and baseline participant characteristics differed. Moreover, this is a very heterogenous group of cancer patients and does not take into account the spectrum of illness of the patients included in the cancer subgroup; for example, both patients with late-stage metastatic disease and patients with early-stage cancers are included in the cancer subgroup. As this is a subgroup analysis of phase 3 trial data, the results may not be representative of a real-world population.

In conclusion, treatment with bezlotoxumab appears to reduce the rate of rCDI compared with placebo and to improve other CDI-related outcomes in participants with cancer. Although these results are encouraging, further research and real-world data are needed to confirm the efficacy of bezlotoxumab in preventing rCDI in individuals with cancer as a comorbid condition.

## Supplementary Data

Supplementary materials are available at *Open Forum Infectious Diseases* online. Consisting of data provided by the authors to benefit the reader, the posted materials are not copyedited and are the sole responsibility of the authors, so questions or comments should be addressed to the corresponding author.

ofaa038_suppl_Supplementary_Material

## Notes

***Financial support. ***This work was supported by Merck Sharp & Dohme Corp., a subsidiary of Merck & Co., Inc., Kenilworth, NJ, USA.

***Writing support. ***Medical writing support, under the direction of the authors, was provided by Ceilidh McConnachie, MSc, of CMC AFFINITY, a division of McCann Health Medical Communications Ltd, Glasgow, UK, in accordance with Good Publication Practice (GPP3) guidelines.

***Potential conflicts of interest. ***O.A.C. has received research grants from, is an advisor to, or received lecture honoraria from Actelion, Allecra Therapeutics, Amplyx, Arsanis, Astellas, AstraZeneca, Basilea, Bayer, Cidara, Da Volterra, F2G, Gilead, GSK, IQVIA, Janssen, Leeds University, Matinas, The Medicines Company, Medpace, Melinta, Menarini, MSD, Miltenyi, Paratek, Pfizer, PSI, Rempex, Roche, Sanofi Pasteur, Scynexis, Seres, Summit, Tetraphase, and Vical. K.M.M. has been an investigator in clinical trials for Astellas, Chimerix, ContraFect, Crestovo, Gilead, GSK, Leonard Merlin Biopharm Inc, MedImmune, MSD, Sage, Shire, and Scynexis, has been on the advisory committee for Chimerix, GSK, MSD, and Scynexis, has been a speaker for Astellas and MSD, and is a DSMB member at Nohla. T.B. is a paid investigator in this study. S.H.S. has been an investigator with Ventura Clinical Trials for Boehringer Ingelheim, Merck, Astellas, Amgen, Seres, Crestovo, Novartis, Pfizer, GSK, Summit, Ablynx, Ironwood, Genentech, Rhythm, Kowa, Shionogi, Diclo, Perrigo, Clinipace, Furiex, Pozen, Roche, Salix, Theravance, Hutchison, Biopharma, Topstone, PSI, ACE, Targacept, Tranzyme, Forest, Alcresta, Baxalta, DalCor, Esperion, Genfit, Gilead, Nichi-Iko, Pioneer, Robarts, Prometheus, Viking, Madrigal, Finch, Bayer, Eli Lilly, Axsome, Genkyotex, CymaBay, Opal, Allakos, and Sage and is a speaker for the World Congress of Digestive Disease. R.N. has nothing to disclose. E.B. has participated in clinical trials and advisory boards sponsored by MSD, Pfizer, and Astellas. D.P.C. has received research funding for his institution, Weill Cornell Medicine, for participating in this research. M.C.E., M.W., and M.B.D. are employees of Merck Sharp & Dohme Corp., a subsidiary of Merck & Co., Inc., Kenilworth, NJ, USA, and may own stock and/or stock options in Merck & Co., Inc., Kenilworth, NJ, USA. All authors have submitted the ICMJE Form for Disclosure of Potential Conflicts of Interest. Conflicts that the editors consider relevant to the content of the manuscript have been disclosed.

***Author contributions.*** O.A.C., E.B., and M.B.D. were involved in the design of this analysis. O.A.C., M.C.E., M.W., and M.B.D. were involved in the analysis of data. O.A.C., K.M.M., T.B., S.H.S., R.N., E.B., and D.P.C. enrolled participants with cancer into the MODIFY I and II trials. All authors were involved in drafting and revising this manuscript and provided final approval of the version to be published. All authors vouch for the accuracy of the content included in the final manuscript. Mary Beth Dorr is the guarantor, and all authors had access to the data.
