# Supplementary material for: Exploratory Evaluation of Bezlotoxumab on Outcomes Associated With Clostridioides difficile Infection in MODIFY I/II Participants With Cancer
Source: Open Forum Infect Dis. 2020 Jan 31;7(2):ofaa038. doi: 10.1093/ofid/ofaa038 (PMC7029680; doi:10.1093/ofid/ofaa038)
Supplement: ofaa038_suppl_Supplementary_Material [file ofaa038_suppl_supplementary_material.docx]

**Supplementary materials**

# Supplementary Table 1. Demographics and clinical characteristics among participants with a solid tumor or hematologic malignancy (mITT population)

|  | Bezlotoxumab | | Placebo | |
| --- | --- | --- | --- | --- |
|  | Solid tumor  N=143  n (%) | Hematologic malignancy  N=53  n (%) | Solid tumor  N=147  n (%) | Hematologic malignancy  N=54  n (%) |
| Clinical characteristics  Inpatient at randomization  Female  Mean age, years (SD)  ≥65 years of age  ≥1 CDI episodes in past 6 months  Severe CDI (Zar score ≥2)^a^  Immunocompromised^b^  Antibiotic during CDI treatment^c^  Antibiotic after CDI treatment^c^  ≥1 predefined risk factors^d^  Charlson Comorbidity Index ≥3  Albumin <2.5 g/dL | 109 (76.2)  69 (48.3)  68.0 (14.6)  94 (65.7)  46 (32.2)  27 (18.9)  33 (23.1)  57 (39.9)  56 (39.2)  123 (86.0)  109 (76.2)  22 (15.4) | 46 (86.8)  30 (56.6)  57.3 (16.9)  18 (34.0)  8 (15.1)  11 (20.8)  50 (94.3)  38 (71.7)  32 (60.4)  53 (100.0)  29 (54.7)  9 (17.0) | 108 (73.5)  79 (53.7)  67.7 (12.0)  91 (61.9)  32 (21.8)  34 (23.1)  29 (19.7)  57 (38.8)  42 (28.6)  114 (77.6)  113 (76.9)  30 (20.4) | 43 (79.6)  21 (38.9)  66.3 (14.4)  32 (59.3)  19 (35.2)  13 (24.1)  46 (85.2)  27 (50.0)  29 (53.7)  52 (96.3)  32 (59.3)  9 (16.7) |
| Anti-CDI antibiotic  Metronidazole  Vancomycin  Fidaxomicin | 72 (50.3)  66 (46.2)  5 (3.5) | 28 (52.8)  22 (41.5)  3 (5.7) | 64 (43.5)  76 (51.7)  7 (4.8) | 22 (40.7)  28 (51.9)  4 (7.4) |
| PCR ribotype^e^  # Participants with a positive stool  027, 078, or 244 strain  027 strain | 100  29 (29.0)  26 (26.0) | 28  4 (14.3)  3 (10.7) | 96  29 (30.2)  26 (27.1) | 38  15 (39.5)  13 (34.2) |

^a^Based on the following: (1) age >60 years (1 point); (2) body temperature >38.3^0^C (>100^0^F) (1 point); (3) albumin level <2.5 g/dL (1 point); (4) peripheral WBC count >15,000 cells/mm^3^ within 48 hours (1 point); (5) endoscopic evidence of pseudomembranous colitis (2 points); and (6) treatment in an intensive care unit (2 points); ^b^defined on the basis of a participant’s medical history or use of immunosuppressive therapy; ^c^systematically bioavailable antibiotics not used to treat CDI; ^d^predefined risk factors for recurrence of CDI: CDI history in the past 6 months, severe CDI at baseline (Zar score ≥2), age ≥65 years, CDI due to hypervirulent strain (ribotypes 027, 078, or 244) and/or immunocompromised; ^e^denominator is participants in the mITT population with a positive culture

CDI, *Clostridioides difficile* infection; mITT, modified intent-to-treat; PCR, polymerase chain reaction; SD, standard deviation; WBC, white blood cell

# Supplementary Table 2. Time to resolution, severity, and treatment of rCDI episode (Clinical Cure population who experienced rCDI during the 12-week follow-up period)

|  | Bezlotoxumab | | Placebo | |
| --- | --- | --- | --- | --- |
|  | Cancer  N=26  n (%) | No cancer  N=103  n (%) | Cancer  N=42  n (%) | No cancer  N=164  n (%) |
| Maximum number of loose stools  during CDI episode  Median  IQR  Min – Max  Time to resolution of new episode (days)  ≤2  3–4  ≥5 | 4.5  3–6  3–10  15 (57.7)  4 (15.4)  7 (26.9) | 4  3–6  3–22  61 (59.2)  20 (19.4)  22 (21.4) | 5  4–7  3–15  18 (42.9)  16 (38.1)  8 (19.0) | 6  4–8  3–23  80 (48.8)  43 (26.2)  41 (25.0) |
| Severity of new episode  Zar score ≥2^a^  Zar score <2^a^  Unknown | 1 (3.8)  20 (76.9)  5 (19.2) | 8 (7.8)  84 (81.6)  11 (10.7) | 8 (19.0)  27 (64.3)  7 (16.7) | 12 (7.3)  133 (81.1)  19 (11.6) |
| New episode treated  Vancomycin  Metronidazole  Fidaxomicin | 11 (42.3)  8 (30.8)  5 (19.2)  1 (3.8) | 64 (62.1)  39 (37.9)  27 (26.2)  6 (5.8) | 30 (71.4)  25 (59.5)  11 (26.2)  2 (4.8) | 106 (64.6)  80 (48.8)  29 (17.7)  8 (4.9) |

^a^Based on the following: (1) age >60 years (1 point); (2) body temperature >38.3^0^C (>100^0^F) (1 point); (3) albumin level <2.5 g/dL (1 point); (4) peripheral WBC count >15,000 cells/mm^3^ within 48 hours (1 point); (5) endoscopic evidence of pseudomembranous colitis (2 points); and (6) treatment in an intensive care unit (2 points)

CDI, *Clostridioides difficile* infection; IQR, interquartile range; mITT, modified intent-to-treat; rCDI, recurrent *Clostridioides difficile* infection; WBC, white blood cell

# Supplementary Table 3. Summary of adverse events (APaT population) and rehospitalization within 30 days of hospital discharge (mITT population who were hospitalized at the time of randomization) in participants with a solid tumor or hematologic malignancy

|  | Bezlotoxumab | | Placebo | |
| --- | --- | --- | --- | --- |
|  | Solid tumor, n (%) | Hematologic malignancy, n (%) | Solid tumor, n (%) | Hematologic malignancy, n (%) |
| Participants in population | 143 | 54 | 147 | 55 |
| During the 24 hours after infusion | | | | |
| Infusion-specific adverse reaction^a^  Discontinued infusion due to adverse reaction | 7 (4.9)  0 (0.0) | 1 (1.9)  0 (0.0) | 5 (3.4)  0 (0.0) | 3 (5.5)  0 (0.0) |
| During the four weeks after infusion | | | | |
| With one or more AEs  With drug-related AEs^b^  With serious AEs  With serious drug-related AEs  Who died^c^ | 96 (67.1)  14 (9.8)  44 (30.8)  1 (0.7)  7 (4.9) | 37 (68.5)  6 (11.1)  14 (25.9)  1 (1.9)  2 (3.7) | 94 (63.9)  8 (5.4)  46 (31.3)  0 (0.0)  10 (6.8) | 44 (80.0)  2 (3.6)  17 (30.9)  0 (0.0)  4 (7.3) |
| Most common AE^d^ | | | | |
| Abdominal pain  Diarrhea  Nausea  Vomiting  Pyrexia  Urinary tract infection  Headache | 9 (6.3)  8 (5.6)  9 (6.3)  6 (4.2)  7 (4.9)  10 (7.0)  6 (4.2) | 3 (5.6)  5 (9.3)  4 (7.4)  5 (9.3)  6 (11.1)  2 (3.7)  1 (1.9) | 6 (4.1)  8 (5.4)  8 (5.4)  3 (2.0)  3 (2.0)  8 (5.4)  5 (3.4) | 5 (9.1)  2 (3.6)  5 (9.1)  2 (3.6)  3 (5.5)  2 (3.6)  1 (1.8) |
| During the 12 weeks after infusion | | | | |
| Serious AE  With sepsis^e^  Who died^e^ | 60 (42.0)  9 (6.3)  15 (10.5) | 23 (42.6)  7 (13.0)  5 (9.3) | 64 (43.5)  11 (7.5)  23 (15.6) | 30 (54.5)  4 (7.3)  8 (14.5) |
| Participants who were hospitalized at the time of randomization^b^ | 109 | 46 | 108 | 43 |
| Any rehospitalization  Associated with CDI | 39 (35.8)  6 (5.5) | 14 (30.4)  2 (4.3) | 34 (31.5)  11 (10.2) | 13 (30.2)  54 (11.6) |

^a^AEs reported on the day of or day after infusion assessed by the investigator to be related to the study infusion. The investigator was unaware of the study-group assignments; ^b^assessed by the investigator to be related to the drug; ^c^rehospitalization and mortality were estimated at 30 days follow-up; ^d^incidence ≥4% in either treatment group reported during the first four weeks after infusion; ^e^sepsis and mortality were estimated at 90 days follow-up

AE, adverse event; APaT, all patients as treated; CDI, *Clostridioides difficile* infection; mITT, modified intent-to-treat

# Supplementary Figure 1. Disposition of participants


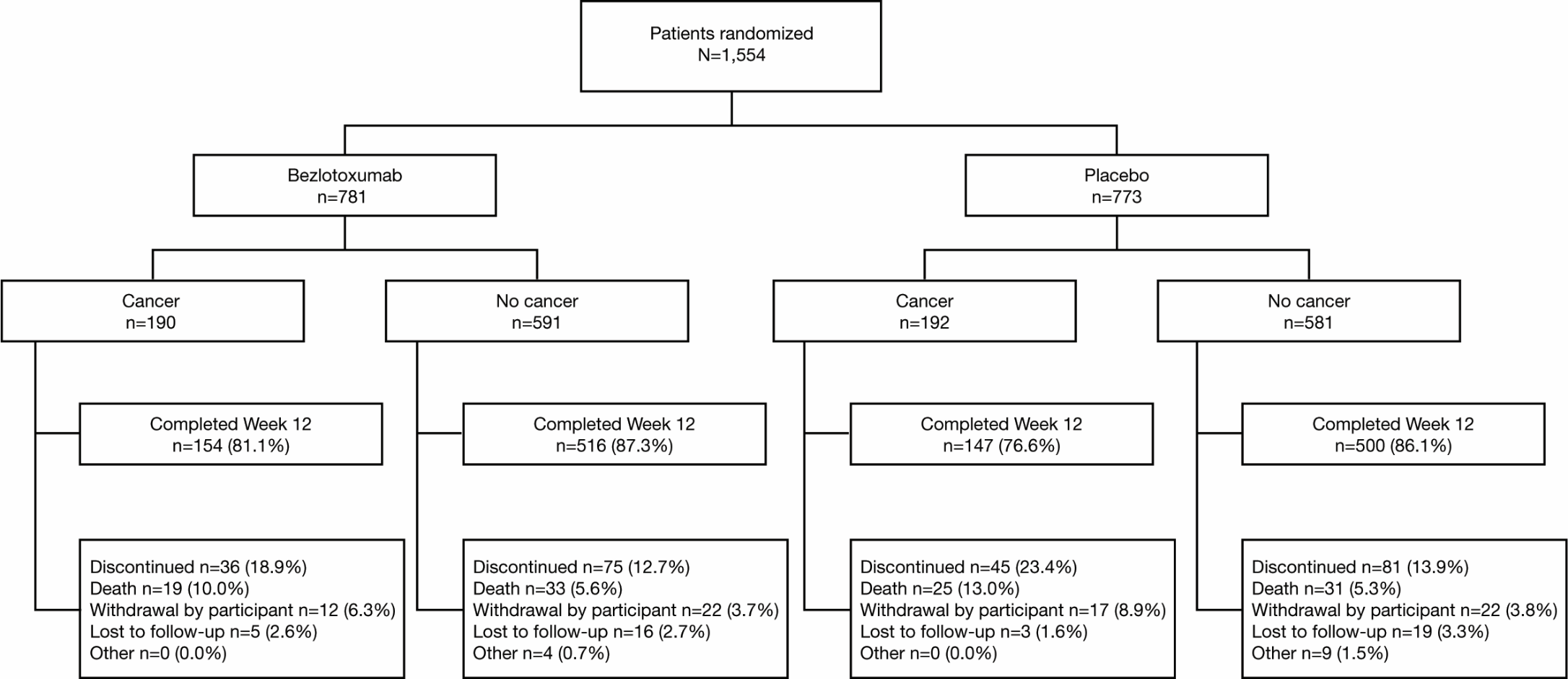


# Supplementary Figure 2. Proportion of participants with ICC, rCDI, and SCC stratified by treatment group in participants with A) solid tumor and B) hematologic malignancy


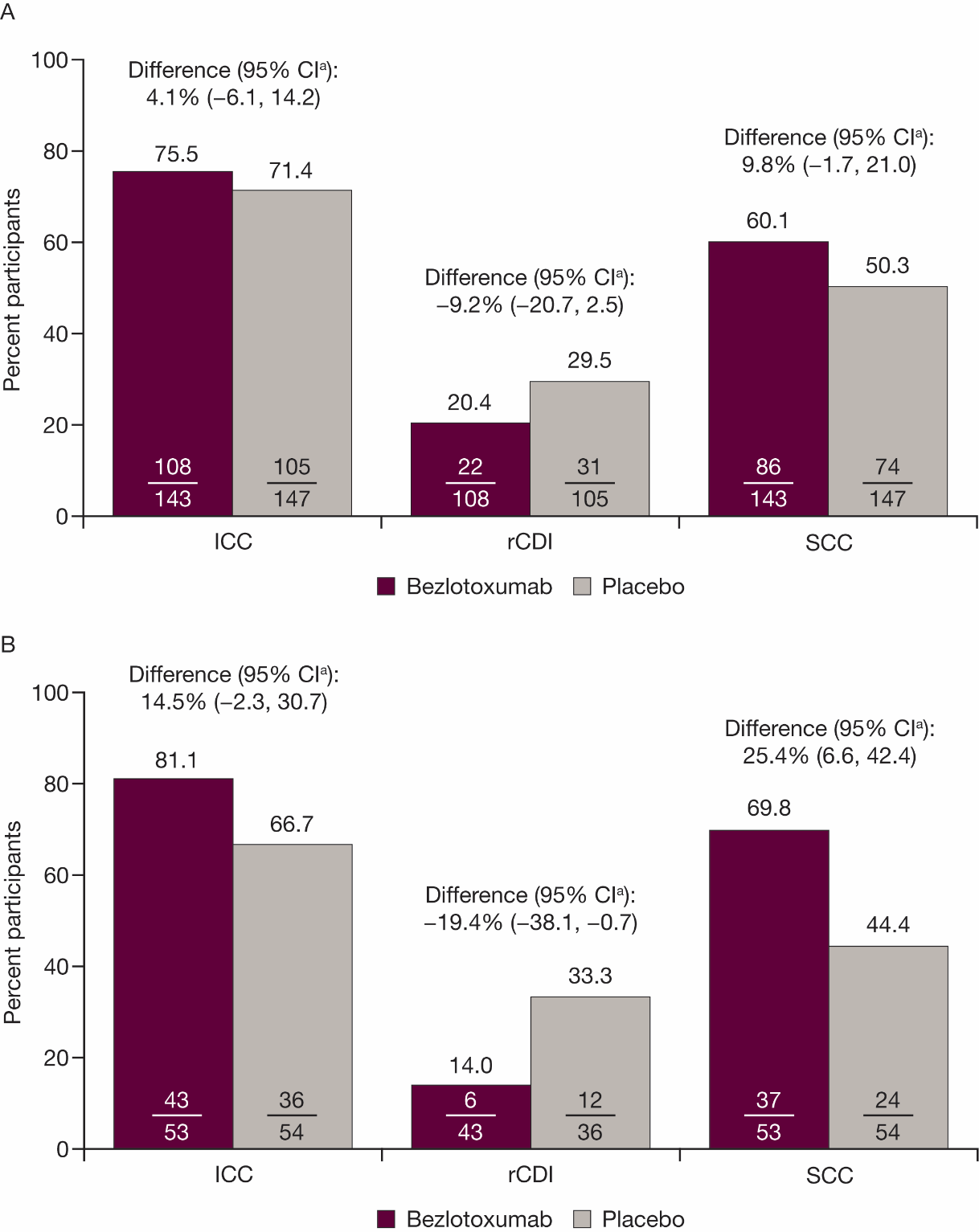


^a^Based on the Miettinen and Nurminen method^1^

CDI, *Clostridioides difficile* infection; CI, confidence interval; ICC, initial clinical cure; rCDI, recurrent *Clostridioides difficile* infection; SCC, sustained clinical cure

**Supplementary Figure 3.** TTROD among participants who received infusion 0–2 days after onset of antibiotic treatment stratified using the log-rank test (mITT population). The start date of resolution of baseline CDI episode was the first of two consecutive days with ≤2 loose stools. Participants who reached the end of their standard of care window (≤16 calendar days) without documented resolution of the baseline CDI episode were censored at the last date of anti-CDI therapy within the window. For participants who were lost to follow-up prior to resolution of the baseline CDI episode, time to event was considered right censored at the date of the last stool record within the anti-CDI therapy window


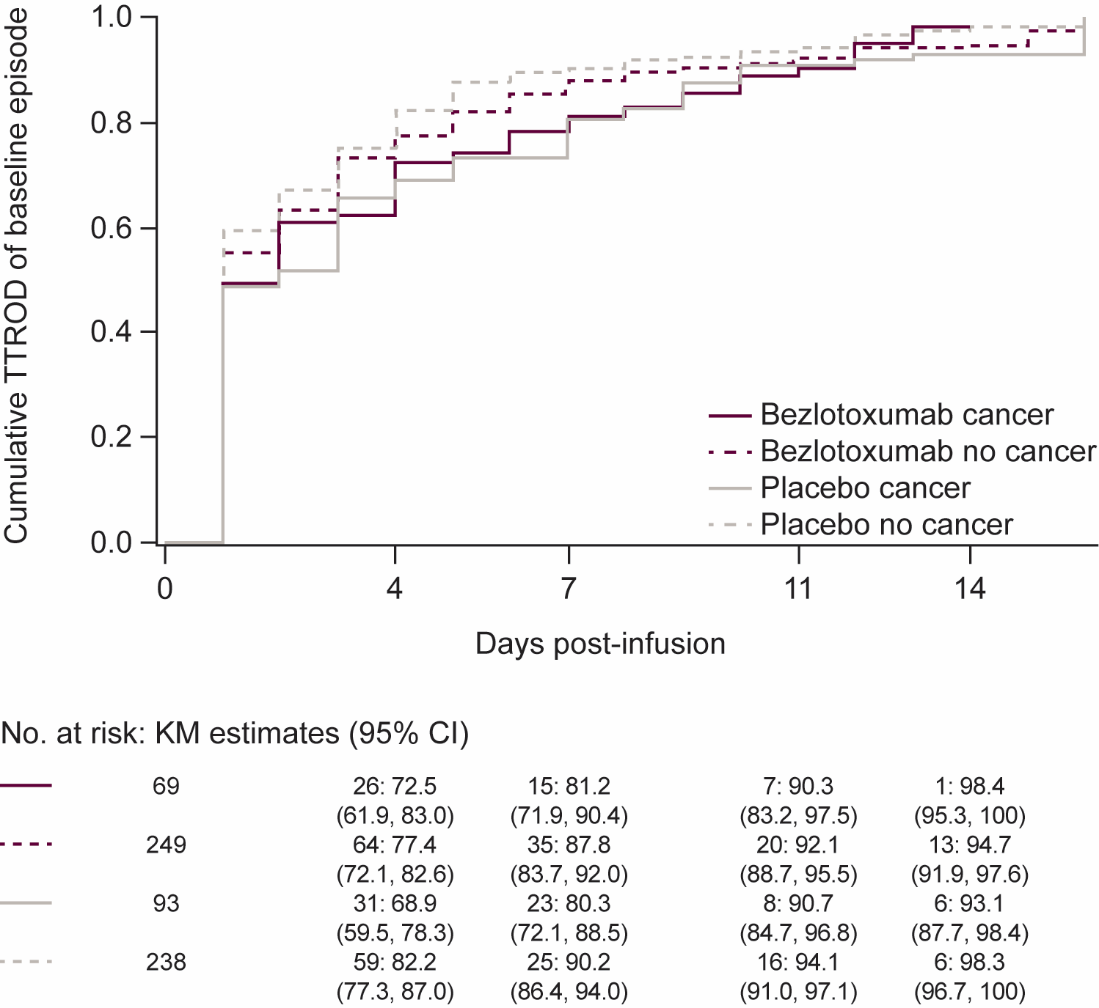


CDI, *Clostridioides difficile* infection; CI, confidence interval; KM, Kaplan-Meier; mITT, modified intent-to-treat; TTROD, time to resolution of diarrhea

# Supplementary Figure 4. Time to death (APaT population)


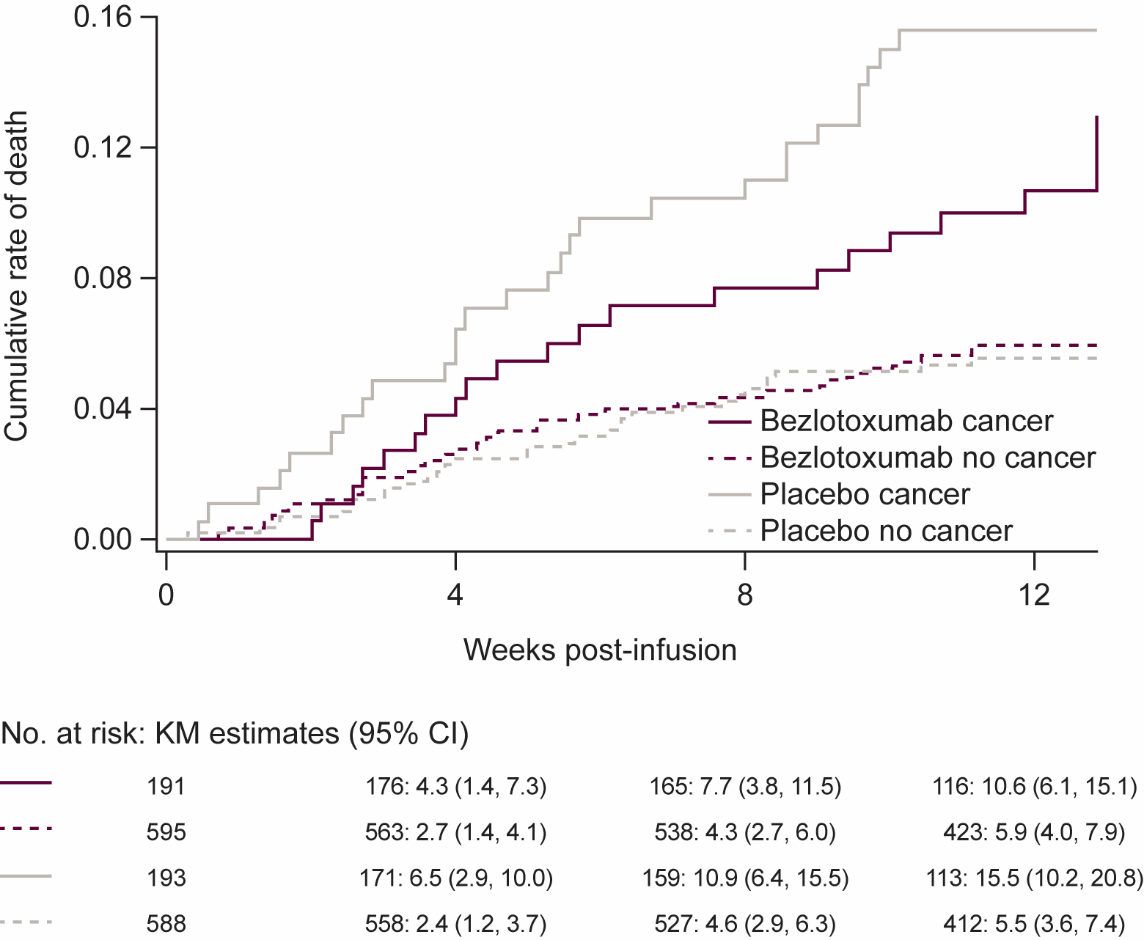


APaT, all patients as treated; CI, confidence interval; KM, Kaplan-Meier

1. Miettinen O, Nurminen M. Comparative analysis of two rates. *Stat. Med.* 4/1985 1985;4(2):213-226.
